# Oral Pharmacokinetics of Hydroxycinnamic Acids: An Updated Review

**DOI:** 10.3390/pharmaceutics14122663

**Published:** 2022-11-30

**Authors:** Kleyton Santos Veras, Flávia Nathiely Silveira Fachel, Bibiana Verlindo de Araújo, Helder Ferreira Teixeira, Letícia Scherer Koester

**Affiliations:** Programa de Pós-Graduação em Ciências Farmacêuticas, Universidade Federal do Rio Grande do Sul, Avenida Ipiranga, 2752, Porto Alegre 90610-000, Rio Grande do Sul, Brazil

**Keywords:** phenolic acids, oral absorption, oral pharmacokinetics, water solubility, stability, intestinal permeability

## Abstract

Hydroxycinnamic acids (HCAs) such as caffeic acid (CA), chlorogenic acid (CGA), coumaric acid (COA) isomers, ferulic acid (FA) and rosmarinic acid (RA) are natural phenolic acids with widespread distribution in vegetal foods and well-documented pharmacological activities. However, the low bioavailability of HCAs impairs their administration by the oral route. The present review addresses new findings and important factors/obstacles for their oral administration, which were unexplored in the reviews published a decade ago concerning the bioavailability of phenolic acids. Based on this, the article aims to perform an updated review of the water solubility and gastrointestinal stability of HCAs, as well as describe their oral absorption, distribution, metabolism and excretion (ADME) processes by in vitro, ex vivo, in situ and in vivo methods.

## 1. Introduction

**Figure 1:**
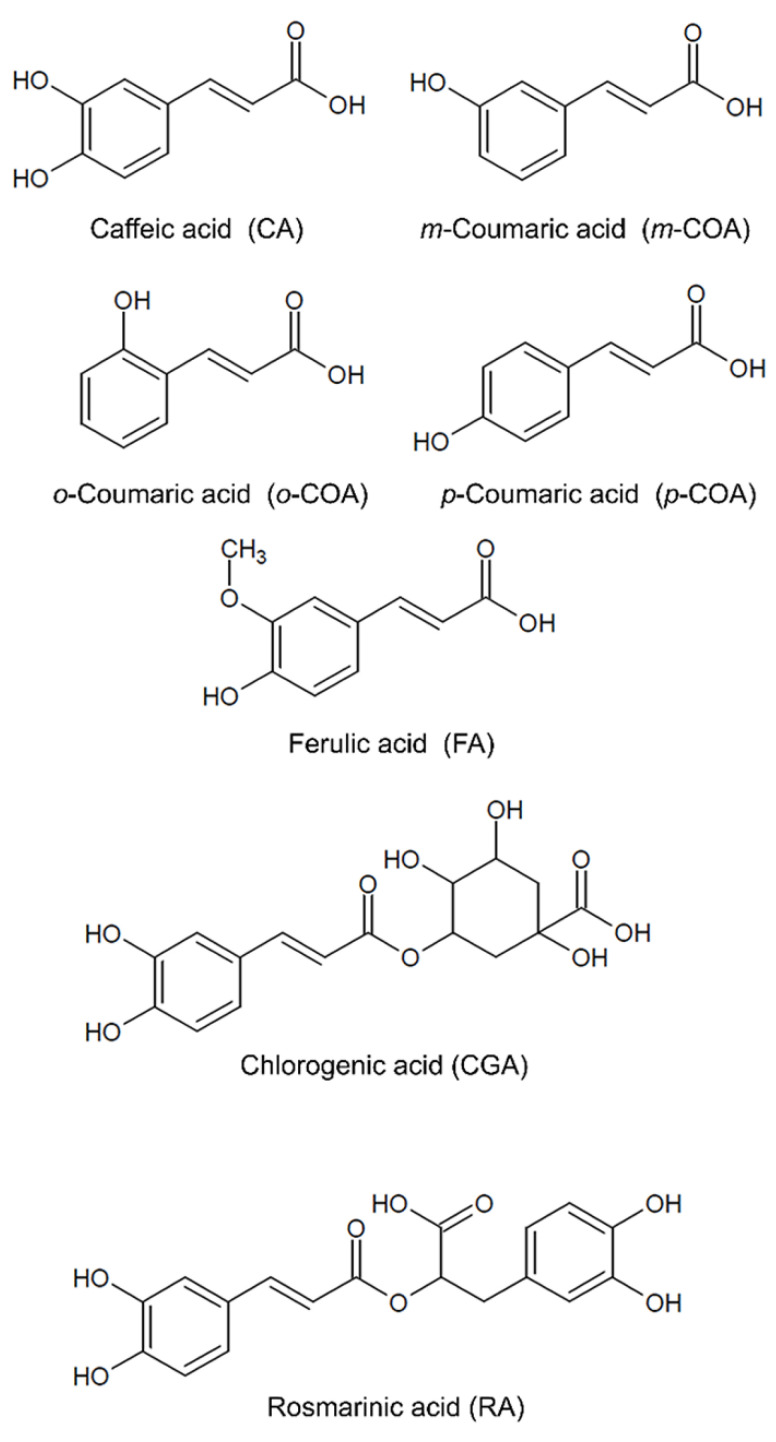
Chemical structure of hydroxycinnamic acids (HCAs).

Hydroxycinnamic acids (HCAs) are a large class of natural phenolic compounds that present cinnamic acid as the basic chemical structure. The HCA class originates from the hydroxylation, methylation, or esterification of cinnamic acid with quinic acid or 3,4-dihydroxyphenillatic acid and is represented by caffeic acid (CA), chlorogenic acids (CGAs), *meta* (*m-*), *ortho* (*o-*) and *para* (*p*-) coumaric acid (COA), ferulic acid (FA) and rosmarinic acid (RA) (Figure 1). CGAs can be subdivided into caffeoylquinic acids, feruloylquinic acids and dicaffeoylquinic acids, of which 5-caffeoylquinic acid is frequently referred to as the main CGA [1,2,3]. 

HCAs have a wide distribution in the food consumed globally, including coffee, food herbs, red wine and whole grains, which have been related to improving the quality of health [4,5,6,7,8]. Additionally, their isolated forms have gained interest in the pharmaceutical field due to their countless pharmacological activities demonstrated by in vitro and in vivo studies, such as analgesic, antibacterial, anti-cancer, anti-diabetic, antifungal, anti-hyperlipidemic, anti-hypertension, anti-inflammatory, antimutagenic, anti-obesity, antioxidant, anti-tyrosinase, immunomodulatory, neuroprotective and photoprotective. On the other hand, their positive effects after oral intake depend upon their bioavailability in the body [5,7,9,10,11,12,13].

Reviews reporting the oral bioavailability of HCAs are available in the literature. Despite the richness of outcomes related to these reviews, important factors for the overall oral bioavailability of HCAs, such as water solubility, stability and physicochemical properties are not fully explored in general. In addition, the reviews do not offer a clear division of the type of approaches applied in the studies, and new findings have been published in the last 10 years [13,14,15].

In this context, this review addresses studies related to aqueous solubility, stability and the oral absorption, distribution, metabolism and elimination (ADME) processes of HCAs evaluated by in vitro, ex vivo, in situ, and in vivo methods. The data are arranged separately when appropriate, according to the factor evaluated and the method applied, in order to create an easy assessment that could be used as a guide for future research targeting the oral administration of HCAs. Studies published before 2010 years have only been described where they are lacking in previously published reviews or where they contain relevant results for the current context.

## 2. Water Solubility of Hydroxycinnamic Acids (HCAs)

Water solubility plays a major role in the oral administration of drugs since it must be in solution to be absorbed in the gastrointestinal tract [16]. Drugs that present water solubility lower than 0.1 mg/mL are classified as poorly soluble according to Horter and Dressmann [17]. Based on the data ascribed in Table 1, all HCAs exhibited water solubility values higher than 0.1 mg/mL, characterizing them as water-soluble compounds. However, it is important to take into account that the strength of the highest drug product should be soluble in 250 mL or less of aqueous media over a pH range of 1 to 6.8 at 37 °C ± 1 °C for a drug substance to be considered highly soluble [18]. Unfortunately, HCAs are not considered pharmaceutical drugs and do not have a defined dose, preventing their Biopharmaceutical classification (BCS), but CA, CGA and RA have been investigated in clinical trials in doses of 300, 200–560 and 10–150 mg, respectively [19,20,21,22,23,24,25,26,27].

CA and FA are the HCAs with more available water solubility data, and some contrasting results have been reported for them. The respective water solubilities of CA and FA at pH 7.2 and 7.4 were stated to be 6.510 and 0.178 mg/mL, and 6.630 and 5.420 mg/mL, respectively, showing a difference of 36.57- and 1.22-fold for a narrow pH range [28,29]. Furthermore, Rastogi and Jana [28] related a higher water solubility for CA at pH 6.8 (0.188 mg/mL) than at pH 7.4 (0.178 mg/mL), when the opposite would be expected (Table 1) since water solubility is a pH-dependent physicochemical property that will increase with a rise in pH for acid compounds, such as HCAs, because of the ionization set by the pKa [30]. 

More pharmacokinetic and pharmacodynamic studies are necessary in order to establish the therapeutic doses of HCAs and classify them into the BCS to estimate the impact of their water solubility on oral bioavailability, which remains an open factor.

**Table 1:** Physicochemical properties of HCAs.

HCA	MW (g/mol)	Water Solubility (mg/mL) ^a^	pKa	Log P	Log D (pH 7.4)	PSA (Å)	HBD	HBA	RB	References
CA	180	0.178 (pH 7.4) 0.188 (pH 6.5) 0.300 (25 °C) 0.420 (pH 3) 0.550 (pH 3.42/15 °C) 0.980 (pH 3.37/25 °C) 1.230 (pH 3.34/30 °C) 1.770 (37 °C) 2.040 (pH 3.25/40 °C) 2.920 (pH 3.17/50 °C) 6.510 (pH 7.2)	4.36–4.70 7.60–9.46 11.17–11.85	0.93–4.60	−1.78–1.74	77.75–81.00	3	4	2	[28,29,31,32,33,34,35,36,37,38,39,40,41]
CGA	354	40.000 (25 °C)	3.33 7.8–8.26	−0.45	−3.91–3.57	164.74	6	9	5	[31,40,42,43,44,45,46]
*m-*COA	164	1.540 (25 °C)	4.48–4.60 10.35–10.39	1.83	−1.34	57.53	2 ^b^	3 ^b^	2 ^b^	[38,39,47,48,49]
*o-*COA	164	0.490 (25 °C)	4.00–4.13 9.58–9.60	1.5	N.A.	57.53	2	3	2 ^b^	[47,48,49,50]
*p-*COA	164	0.344 (25 °C) 0.700 (37 °C)	4.36–4.70 8.84–9.92	1.43–4.45	−1.32	56.20–57.50	2	3	2	[32,33,38,40,47,51,52]
FA	194	0.333 (25 °C) 0.454 (25 °C) 0.570 (pH 3.54/15 °C) 0.710 (pH 3) 0.780 (pH 3.46/25 °C) 0.920 (pH 3.40/30 °C) 0.950 (pH 3.40/30 °C) 1.490 (37 °C) 1.760 (pH 3.31/40 °C) 2.190 (pH 3.18/50 °C) 5.420 (pH 7.4) 6.630 (pH 7.2)	4.58–4.96 9.68–9.99	1.25–4.12	−1.38–1.23	66.80	2	4	3	[29,32,33,36,38,39,40,41,50,51,52,53,54]
RA	360	1.800 (pH 1.2/ 25 °C)	2.92 8.36 9.56 10.62	1.63–1.78	−2.45	144.52–145.00	5	8	7	[39,55,56,57,58]

^a^ Some studies did not relate the pH or temperature used in the solubility assay. ^b^ Data not available in the literature and obtained from SwissADME software [59]. N.A. not available in the literature or SwissADME software. MW: Molecular weight. Log P: partition coefficient. log D: distribution coefficient. PSA: polar surface area. HBD: number of hydrogen bond donor. HBA: number of hydrogen bond acceptor. RB: rotatable bonds.

## 3. Stability of Hydroxycinnamic Acids (HCAs)

Stability in simulated gastrointestinal medium and plasma are assays that allow the loss of content catalyzed by the environmental conditions to be estimated [60,61]. Simulated gastrointestinal stability studies for CA and RA, in isolated form, were performed in two phases: gastric and intestinal [62,63,64,65,66]. The studies demonstrated that CA and RA present a content recovery higher than 98% after the gastric phase, while the recovered fraction decreased to 46–75% and 69–75%, respectively, in intestinal conditions [62,64]. CGA exhibited stability of 48% after all simulated gastrointestinal assays [67]. The lower content of CA, RA and CGA after the gastrointestinal assay was ascribed to the low chemical stability of phenolic compounds in the alkali medium of the intestinal phase [62,64,67]. FA is stable in the gastric environment [68]; nevertheless, there are no reports about its stability in intestinal conditions. The substantial loss of content could be the first obstacle for the oral delivery of HCAs. 

Conversely, Ren et al. [42] showed that the gastric and intestinal phases chemically affect the stability of CGA, while Costa et al. [65] and Zoric et al. [63] reported a significant loss of RA in the gastric phase when compared with the intestinal phase. The effect of other compounds in the digestive solution on the stability of CGA was also investigated by employing a mixture of three phenolic compounds plus CGA. The CGA content in the mixture composed of cyanidin 3-rutinoside, quercetin-3-rutinoside, catechin and CGA was 94.9%, while for the mixture composed of quercetin, quercetin 3-O-glucoside, rutin and CGA had a value of 11.5% [69,70], suggesting that the matrix had an influence on the stability of CGA.

In addition to evaluating the stability of compounds, the simulated gastrointestinal medium was also employed to analyze the free fraction of CA, CGA and FA from food, since they can interact chemically with amino acids, peptides and proteins. Simulated proteolytic digestibility revealed a low release of HCAs from proteins, a fact that decreases the availability of their free forms for absorption [71]. 

Stability in plasma is crucial to maintain the drug in a desirable concentration in the body and to promote its pharmacological effect [61]. The stability of CGA in rat and human plasma and phosphate buffer (pH 7.4) solution at 37 °C revealed the formation of a substance with a similar fragmentation pattern to CGA, identified as its isomer. The pH-dependent isomerization reaction of CGA is well-described in the literature; thus, it is more probable that its isomerization was due to the alkali pH rather than an enzymatic reaction [72,73]. RA was stable in rat plasma and phosphate buffer at 37 °C, while a de-esterification reaction occurred in human plasma, forming CA (<4%) [72]. 

Despite the absence of specific plasma stability analyses for CA, *p-*COA and FA in the literature, bioanalytical validation studies indicated that CA was 70% stable in rabbit plasma for 24 h at 25 °C [74], and *p-*COA and FA were stable for 4 h and 24 h, respectively, in rat plasma at room temperature [75,76]. Studies relating to the gastrointestinal stability of COA isomers and the plasma stability of *m*- and *o-*COA isomers were not found in the literature.

## 4. Absorption, Distribution, Metabolism and Elimination (ADME) of Hydroxycinnamic Acids (HCAs)

### 4.1. In Vitro Studies

#### 4.1.1. Absorption

In vitro methods are extensively used in the screening of drugs, aiming to elucidate their oral absorption mechanism and the fraction captured or permeated, presenting advantages over in vivo methods, such as lower cost and faster analysis. Artificial membrane-based and cell culture-based methods are among the in vitro methods used, such as the parallel artificial membrane permeability assay (PAMPA), and 2/4/A1, Caco-2, IEC-18, HT29-MTX, MDCK, T7 cell lines, and Caco-2/HT29-MTX, Caco-2/Raji B, Caco-2/HT29/Raji B co-cultures, respectively [77,78,79].

PAMPA is a method that mimics the transcellular passive intestinal permeability of drugs and enables their diffusion in different types of lipids and pH to be estimated [80]. Apparent permeability coefficient (P_app_) values higher than 10.0 × 10^−6^ cm/s, between 5.0 and 10.0 × 10^−6^ cm/s and lower than 5.0 × 10^−6^ cm/s classify a drug as highly, moderately and slowly absorbed, respectively [28].

The PAMPA of CA at three concentrations indicated a P_app_ value lower than 4 × 10^−6^ cm/s [28]. In another study, an even lower P_app_ value, expressed as the logarithm of P_app_ (log P_app_ = −5.52 or P_app_~3 × 10^−6^ cm/s), was found for CA, CGA and RA in the pH range 4.0–8.0. These P_app_ values mean that less than 1% of each HCA was transported toward the receptor compartment [81,82,83]. A higher transport rate was only observed for FA at pH 4.0, for which the log P_app_ was −4.77 (P_app_~17 × 10^−6^ cm/s); over pH 4.0, the P_app_ values were equal to the other HCAs [81,82]. 

**Figure 2:**
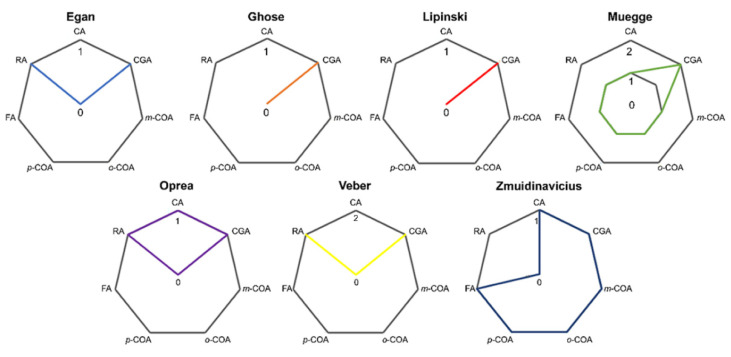
Number of violations for HCAs in each prediction rule.

Transcellular passive diffusion is a process affected by the physicochemical properties of the drug, including the molecular weight (MW), partition coefficient (log P), distribution coefficient (log D), number of hydrogen bond donors (HBDs) and acceptors (HBAs), pKa, number of rotatable bonds (RBs) and polar surface area (PSA) [77,81,82,84]. Based on the different physicochemical properties, some drug oral absorption prediction rules are used in medicinal chemistry research and the pharmaceutical industry, such as the rules of Egan et al. [85], Ghose et al. [86], Lipinski et al. [87], Muegge et al. [88], Oprea [89], Veber et al. [90] and Zmuidinavicius et al. [91], and the HCAs violate them in one or more parameters (Figure 2).

CGA violates all prediction rules, displaying the highest number of violations, followed by RA > CA > FA. FA appears to be the most favorable HCA to permeate by transcellular passive diffusion, which is visualized at pH 4.0 by PAMPA at least. Van de Waterbeemd et al. [92] affirmed that a drug with log D higher than one unit at pH 7.4 (pKa-dependent property) is more easily transported by transcellular passive diffusion. In this sense, the higher P_app_ of FA over the other HCAs is justified, since it presents the highest first pKa and log D 7.4 values (Table 1). The COA isomers demonstrate violations in the prediction rules; however, there are no studies in the literature about their transcellular passive diffusion. 

It is important to point out that the oral absorption of a drug is the sum of passive and carrier-mediated influx and efflux transporters [93]. In this context, the in vitro cell culture-based techniques are more adequate to evaluate the absorption of drugs, since they express influx and efflux transporters. Aside from that, they allow the intestinal and hepatic metabolism by phase 1 and 2 metabolic enzymes to be estimated [94,95].

Permeability studies in the Caco-2 cell line demonstrated that FA, *m-* and *p-*COA in the presence of an inward-direct proton gradient are transported by the transcellular mechanism through monocarboxylate transporters (MCT), with a linear concentration-dependence; in the absence of this proton gradient, their transport occurs by passive diffusion [96,97,98]. CA, CGA and RA are mainly transported by the paracellular mechanism, since they do not have a polarised flux. However, it has been shown that the transport of CA and RA also occurs, to a lesser extent, by transcellular mechanisms through MCT and organic anion-transporting polypeptide (OATP) transporters [59,99,100]. 

The intracellular accumulation of the HCAs was <2%, and the rate of transport, defined as the amount permeated toward the basolateral side, followed the order: FA (3.42–30.52%) > *m*-COA (1.34–13.74%) > *p*-COA (1.55–10.87%) > CA (0.10–1.57%) > RA (0.03–1.30%) > CGA (0.10–0.30%) [59,96,97,98,99,101,102]. The results expressed as P_app_ (apical → basolateral) (cm/s) were similar: FA (10.0 × 10^−6^) > *o*-COA (6.0 × 10^−6^) > *m*-COA = *p*-COA (5.0 × 10^−6^) > CA (1.40–2.12 × 10^−6^) > CGA (0.38–0.86 × 10^−6^) > RA (0.20–0.86 × 10^−6^). These data indicate that FA and COA isomers show the highest permeability when compared to CA, CGA and RA. Variations in the amount permeated and P_app_ is caused by differences in the experiment time or the presence or absence of a proton gradient [28,48,102,103,104,105]. Recently, Mortelé et al. [106] reported a P_app_ of 2.42 × 10^−6^ cm/s for CGA, which is substantially higher than the other values found for this HCA.

Studies performed with vegetal matrices containing CA, *p*-COA and RA showed an increase in their rate of transport, while a negative effect was reported for CGA. The vegetal matrices were composed of numerous substances that have different degrees of affinity by the transporters, which could change the permeability of the HCAs [45,100,102,107,108].

The transport of CA, CGA, *p-*COA and FA was also investigated in other cell lines [45,109,110]. CGA in gastric cells presented an absorption twice as high as in Caco-2 cells, a finding associated with the acid pH of the medium [45]. The paracellular transport is influenced by the charge state of the drug, in which the uncharged state is desirable [111]. Based on the pKa of CGA (Table 1), the acid pH (pH 3.0) of gastric cells maintained more than 50% of CGA in the uncharged state, whereas more than 50% of CGA would be in a negatively charged state in Caco-2 cells (pH 7.4). 

In a Caco-2/HT29-MTX co-culture, a higher amount of FA permeated in the presence of a proton gradient. The transport was linear and independent of concentration, a characteristic of the transcellular passive diffusion mechanism [109], contrasting with the outcomes reported by Konishi and Shimizu [96]. Additionally, in the T84 cell line, among CA, CGA, *p-*COA and FA, only FA was detected on the basolateral side, demonstrating that it is the most permeable HCA and confirming the results shown by the PAMPA and Caco-2 cell methods [81,82,96,110]. 

Efflux transporters, mainly P-glycoprotein (P-gp), multidrug resistance-associated protein 2 (MRP2) and breast cancer resistance protein (BCRP), also have a significant contribution with respect to drug transport [112]. The efflux ratio was lower than two for CA, CGA, FA and RA, suggesting that they are not substrates for efflux transporters [103,105]. Nonetheless, for CGA, this hypothesis is refuted by the use of P-gp, MRP2 and BCRP inhibitors that improve its absorption [106,113]. In addition, CA and RA showed a concentration-dependent inhibitory or inducer activity on P-gp, MRP2 and BCRP [114,115,116,117,118], while FA expressed only an inhibitory activity on P-gp [119,120].

#### 4.1.2. Metabolism

Cytochrome P450 isoenzymes (CYP), catechol-*O*-methyltransferase (COMT), UDP-glucuronosyltransferases (UGTs) and sulfotransferases (SULTs) are the main enzymes present in the cells of the body responsible for drug metabolism. Enzymes present in Caco-2 cells metabolized CA to CA glucuronide, CA methyl-glucuronide, CA sulfate, FA and isoferulic acid. CGA underwent isomerization, hydrolysis (formation of CA), sulfonation and methylation (formation of feruloylquinic acid). *p*-COA was very stable, presenting just traces of its glucuronidated, methylated and sulfated forms. FA produced FA glucuronide, FA sulfate and dihydroferulic acid. These similar metabolites were also detected in the Caco-2/HT29-MTX co-culture and T84 cell line. RA underwent isomerization, hydrolysis (formation of CA) and methylation. The appearance of HCA metabolites after incubation with Caco-2 cells reveals that the intestine would be the first site of their metabolism. Quantitatively, the free forms of CA (69.56–94.6%), CGA (73.1–94.7%), *p-*COA (~100%), FA (~95.50–95.62%) and RA (88.25%) remained at a higher concentration than the metabolites [101,104,109,110,121,122,123]. 

Data about the metabolism of HCAs in HepG2 cell are available for CA, CGA, FA and RA. The free HCAs were the predominant forms (>63.8%); compared to the Caco-2 cells, extra metabolites were only detected for RA, which was identified as RA glucuronide, RA methyl-glucuronide and FA [122,123]. Moreover, liver microsomes produced 14 metabolites from RA, which were glutathione conjugates and glucuronidated forms [124]. The incubation of CA and FA with isolated hepatocytes and liver microsomes revealed the formation of dihydrocaffeic acid, dihydroferulic acid and CA glutathione conjugates [125,126]. Additionally, in human intestinal and liver S9 homogenates, the predominant pathway of metabolization for CA and FA was sulfation (>95%), while glucuronidation was extremely low [127].

**Figure 3:**
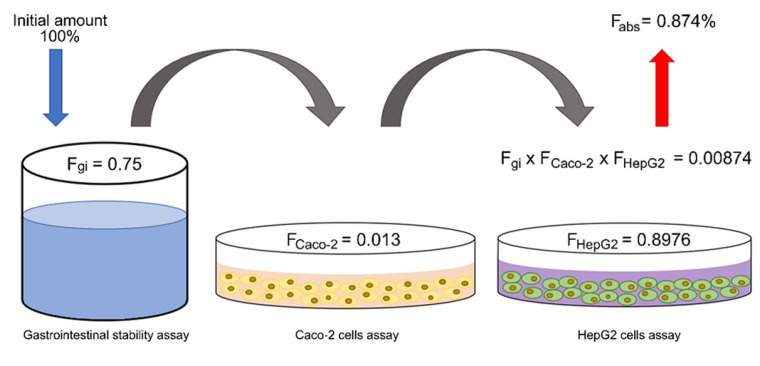
Scheme of processes involved in the oral absorption of rosmarinic acid (RA) and its remaining fraction after gastrointestinal stability (F_gi_), intestinal absorption (F_Caco-2_), hepatic metabolism (F_HepG2_) and total oral bioavailability (F_abs_), evaluated by in vitro methods.

The cell culture-based methods identified a large number of HCA metabolites, which sometimes differed qualitatively and quantitatively, demonstrating that they express distinct metabolization pathways [121,122,123,125,127]. Regardless of the lack of homogeneity in some results, the in vitro methods remain the first choice in elucidating the mechanism involved in the absorption and metabolism of drugs. Figure 3 illustrates a scheme for the oral absorption of RA based on the gastrointestinal stability, Caco-2 cell permeability and HepG2 cell metabolism studies, predicting a bioavailable fraction lower than 1% [62,102,122].

Microbial metabolism in the intestinal tract also has a considerable effect on the available fraction for absorption when the drugs are administered by the oral route. The in vitro fermentation of CA and CGA carried out with human fecal microbiota, revealed that the HCAs evaluated over a time interval of 0.5–2 h were undetectable and the metabolite 3-hydroxyphenylpropionic acid (3-HPPA) was identified for both. The maximum concentration (C_max_) of 3-HPPA was reached at 2 h; afterward, it was also completely degraded [128,129]. Another study reported that the 6 h incubation of CGA with human fecal microbiota produced 11 metabolites; among them, dihydroxycaffeic acid, dihydroxyferulic acid and 3-HPPA comprised 75–83% [130]. 

FA incubated with rat fecal microbiota apparently had a slower degradation during 48 h, forming two metabolites: dihydroxyferulic acid and 3-methoxyl-4-hydroxybenzenepropanoic acid [131]. For RA, the probiotic strain *Lactobacillus johnsonii* isolated from the human intestinal microbiota completely hydrolyzed the RA into CA and 3,4-dihydroxyphenyllactic acid after 4 h [132].

In summary, the in vitro PAMPA and Caco-2 cell experiments indicated that the physicochemical properties of HCAs can limit their absorption across the gastrointestinal membranes, with FA being the most absorbed compound among the HCAs. The effect of efflux transporters on the total absorption of CGA was also confirmed, in addition to its physicochemical properties. On the other hand, microbiota, intestinal and hepatic metabolisms could considerably reduce the systemic amount of HCAs.

### 4.2. Ex Vivo Studies

Compared to the in vitro assays, ex vivo methods provide more distinctive features of the intestinal tissue structure [133]. The rate of transport of CA, CGA and *p*-COA in the porcine intestinal mucosa (cecal pole) was 3.7, 1.9 and 3.3%, respectively [134]. In the porcine jejunal segment, the amount of CA and CGA absorbed was lower than 1.5%. For CGA, the transport kinetic was linear and non-saturable, which is consistent with the data from in vitro methods [99,135]. The recovery of FA in the rat ascending or descending colon was statistically similar, reaching values of 4.66% and 4.76%, respectively [109].

Only CGA and *p*-COA transport were considered in the different segments of the gastrointestinal tract employing the same tissue source, with CGA being more permeable in the duodenum than in the ileum, jejunum and colon, while *p*-COA was transported more in the jejunum and ileum than in the cecum, colon and stomach [136,137]. When the rate of transport was analyzed in tissues from different animal species and intestinal segments, the results were not consistent. This is exemplified by CA, which showed greater transport in porcine ileum, whereas the highest rate in rats was found in the jejunum [134,135,138]. 

An increase in CGA transport was achieved due to the synergic action of the extract components [137] and the use of a P-gp inhibitor [135]. The transport of RA contained in a vegetal matrix was evaluated in rat jejunum mucosa, demonstrating an increase in transport with an increase in the vegetal matrix concentration. Nonetheless, neither the initial RA content nor the relative amount transported is reported [139].

With regard to metabolism, the glucuronidated form of CA was found to be in a higher concentration than the free form [138]. CGA was metabolized to its isomers and CGA glucuronide, and also hydrolyzed to CA [135]. *p-*COA did not present any metabolites, while FA produced the same metabolites as described in the in vitro studies, Section 4.1.2 [109,138]. There are no studies in the literature reporting the use of ex vivo methods to evaluate the absorption of *m-* and *o*-COA isomers.

The quantitative data obtained from ex vivo methods are not comparable, due to the use of different animals and tissue types; most of the studies did not reveal the amount transported relative to the initial dose. Despite this, from the available data, the highest absorption of FA and metabolization of HCAs was perceived in accordance with the in vitro methods. 

### 4.3. In Situ Studies

In situ methods offer advantages such as an intact gastrointestinal mucosa, and the presence of a nervous system and blood flow, which are not possible to obtain in the in vitro-based cell and ex vivo studies, getting closer to the in vivo mechanisms [133]. In order to avoid the use of the term recurrent, all in-situ gastric absorption studies described in this section were carried out with the pylorus ligation.

From the gastric route, 89.7% of free CA was measured in the portal vein after 5 min of administration, revealing its fast and considerable absorption. A decline in the concentration of free CA was observed in the abdominal aorta (39.1%), which indicated that CA underwent metabolism in the liver [140]. In contrast, its perfusion in the duodenal and jejunal plus ileal segments showed that only 12.4% and 19.5% of CA was absorbed, respectively, displaying a lower absorbed fraction when compared to the gastric assay, regardless of hepatic metabolism [141,142]. The identification of FA and isoferulic acid in the perfusion samples provides evidence of the metabolism of CA in the passage through the intestinal membranes [142].

Studies for CGA demonstrated that its absorption is independent of concentration, a characteristic of substances transported by passive mechanisms [42,99,137]. When administered in the stomach, 9.43% and 4.57% of CGA were recovered in the gastric vein and aorta in free form, respectively, without the detection of metabolites [143]. Konishi et al. [140] confirmed its low gastric absorption and the reduced concentration in the abdominal aorta, evidencing its hepatic metabolization. In addition, 8% was absorbed through CGA perfusion in the jejunal plus ileal segment, and isoferulic acid was identified as a product of its intestinal metabolism [142]. 

The rate of absorption of CGA was very close in the stomach and intestine: the lack of significant difference between them was posteriorly demonstrated by Ren et al. [42], who reported the respective concentrations of 7.7% and 7.9% for the gastric and intestinal experiments. When assessing the degree of permeability according to intestinal segments, the duodenum was found to be more permeable than the ileum, in accordance with the ex vivo assay [137].

The gastric administration of *p-*COA promoted the absorption of 73.3% in the portal vein and 57.5% of the remaining content in the abdominal artery in the free form. In contrast to the other HCAs, the decrease in the free form was accompanied by the metabolized forms, but the reduction in the latter occurred to a lesser extent [140]. Intestinal in situ experiments were not found for *p*-COA.

Two in situ gastric studies of FA demonstrated its high absorption rate in the free form [68,140]. The amounts collected in the portal vein in the free, glucuronidated, sulfated and sulfoglucuronidated forms were 49.2%, 2.9%, 2.6% and 43.1%, respectively. However, a decrease in the FA-free form (6.2%) was observed in the samples from the celiac artery, while the FA-metabolized forms increased (93.8%), a factor derived from its hepatic metabolism and biliary excretion [68]. This was corroborated by Konishi et al. [140] who reported the presence of 62.8% free FA in the portal vein, while its concentration fell to 18.4% in the abdominal artery. Based on the free FA collected in the urine (4.6%), probable renal metabolism would also be responsible for the concentration decline [68]. It is important to highlight that these two studies revealed a 2.97-fold difference in the amount of free FA after hepatic metabolism [68,140].

In the intestinal perfusion, 60% of FA detected in the mesenteric vein was in the free form and 40% was in the metabolized form. A 2.4-fold reduction was observed in the sample collected in the abdominal aorta, assuring hepatic metabolization [144]. Comparing these results with those reported previously indicates that the absorption of FA is higher in the intestine than in the stomach, and the intestine is the first site of metabolism, followed by the liver [68,140,144].

FA contained in a semipurified sample was transported in the jejunal plus ileal segment in a manner directly proportional to its perfused concentration, presenting a non-saturable mechanism [145]. The rate absorbed in the intestinal segment was 56.1%, and no metabolites were detected after intestinal passage, in contrast with the data previously described. Nevertheless, after metabolism in the liver, no free FA was found in the bloodstream [138,144,145]. This section does not discuss the *m*- and *o*-COA isomers and RA due to the lack of studies in the literature.

The analysis of all in situ data showed that the free fraction of *p*-COA (57.5%) was superior to CA (39.1%), FA (6.2–18.4%) and CGA (4.57%) after hepatic metabolism, denoting the impact of metabolization on the bioavailability of HCAs.

### 4.4. In Vivo Studies

Reviews reporting the oral pharmacokinetics of HCAs within vegetal matrices are already available in the literature [14,15]. Based on this and the finding that other compounds can modify the pharmacokinetic processes of HCAs, as seen in the in vitro and ex vivo assessments, only studies with isolated HCAs were selected for review in this section. The in vivo pharmacokinetic parameter data are provided in Table 2.

#### 4.4.1. Absorption

The in vivo pharmacokinetic data revealed that the HCAs presented rapid absorption after oral administration, with absorption t_1/2_ and T_max_ ranging between 0.07 and 0.08 h and 0.03 and 1.5 h, respectively (Table 2). For the [3–^14^C] CA, the highest plasma radioactivity was visualized at 0–1 h [163]. The short time to reach the C_max_ suggests that HCA compounds, in part, are absorbed in the gastric environment. In fact, the in situ gastric studies described above indicated that more than 60% of CA, *p*-COA and FA were transported to the portal vein after 5 min [140].

The rate of absorption obtained from the area under the curve (AUC)/dose relationship increased in the following order: CGA < RA < *p-*COA < FA < CA (Table 2). This order demonstrates that CA was more absorbed than FA and *p*-COA, data that were not observed in the in vitro, ex vivo and in situ evaluations [96,99,138,142,144]. The measure of AUC is affected by several factors, including the blood-sampling site. A comparative study revealed a significant increase in the AUC value for blood samples collected from the carotid artery compared to the caudal vein [164]. The respective sampling sites that presented the highest AUC for CA and FA in pharmacokinetic studies were the carotid artery and the caudal vein [146,158], a fact that, in part, could justify the higher rate of absorption exhibited for CA. Furthermore, the AUC for *p-*COA and FA was not extrapolated to infinite time [153,158]. The AUC/dose relationship based on the study by Kim et al., (2020) [155] was excluded as the dose was not defined by the weight.

#### 4.4.2. Distribution

The distribution of a drug through the body is dependent on its physicochemical properties and plasma protein binding [165]. Among the HCAs with the available volume of distribution (Vd) data (Table 2), FA presented the highest value, suggesting a wide distribution [158]. In fact, FA exhibited a two-compartmental pharmacokinetic profile, denoting a distribution phase prior to elimination [156]. The Vd values for CA, CGA and RA were clearly lower than FA but also indicated a tissue distribution, since the values were superior to the real animal body volume. Additionally, the t_1/2α_ of CA and FA revealed that their distribution is slower than their absorption when orally administered (Table 2) [141,146,149,156,160,166].

Considering the physicochemical characteristics given in Table 1, FA is the most lipophilic HCA and its plasma binding protein value (73.5%) is higher than CA (66%) and CGA (25.6%) [150,167]. Together, these data reveal that adequate physicochemical properties are more important to the partition toward tissues than only the plasma-free fraction for HCAs. The lower lipophilicity and higher binding protein value (91.4%) of RA could explain its smaller distribution compared to FA [168]. 

The specific tissues reached by CA were the kidneys, liver, muscles, lungs, heart, spleen and testes. The kidney (3.2%) and liver (0.3%) showed the highest concentration of CA, while the rate of CA was equal to or lower than 0.1% for the other tissues [163]. De Oliveira et al. [151] described CGA as being distributed through the kidney, liver and muscles, but quantitative analyses were not performed. Broader and quantitative tissue distribution of CGA was carried out by Chen et al. [152], who observed a decrease in the AUC in the following order: liver > kidney > heart > spleen > lung. For FA, the AUC of the kidney represented 76% of its tissue distribution. Minority AUCs were measured in the liver, lung, heart, spleen and brain [158]. Oral pharmacokinetic studies for *p*-COA and RA did not describe their tissue distribution. 

#### 4.4.3. Metabolism

The metabolism of HCAs is catalyzed mainly by hydrolysis and conjugation by COMT, UGTs and SULTs [148,151,158,159,161,163,169]. A drastic decrease in the AUC and C_max_ measured from the portal vein and the abdominal artery was observed for CA, *p*-COA and RA. The reduction in free RA was accompanied by an increase in its metabolites, indicating additional metabolism by the liver. On the other hand, the concentration of free and metabolized forms of CA and *p*-COA declined simultaneously [147,153]. 

CA was metabolised to CA 3- or 4-*O*-glucuronide, CA 3- or 4-*O*-sulfate, CA sulfoglucuronide, FA, FA 4-*O*-glucuronide, FA 4-*O*-sulfate, isoferulic acid and isoferulic acid 3-*O*-sulfate [148,163,169]. The plasma concentrations of free FA and isoferulic acid were lower than free CA, while its glucuronidated (26 µM) and sulfoglucuronidated (~12 µM) forms had higher plasma concentrations than free CA (1.2 µM) [148,169]. These quantitative results were shown in the ex vivo studies and in the majority of the in vitro assessments [122,123,138].

The CGA metabolites present in the plasma were CGA glucuronide, CA, CA glucuronide, hippuric acid, FA sulfate, feruloylquinic acid isomers and isoferulic acid sulfate. The plasma C_max_ value for the metabolites was 2-fold lower than the free CGA. Other metabolites were also detected in the tissues, such as CA sulfate, dihydrocaffeic acid, FA, FA sulfoglucuronide and isoferulic acid sulfoglucuronide [151]. The preceding pharmacokinetic evaluation did not detect the presence of CGA in the plasma, even in a higher dose, and only three CGA metabolites were identified: CA glucuronide, CA sulfoglucuronide and FA sulfoglucuronide [169]. Compared to the in vitro and ex vivo studies, the in vivo assays distinguished a higher number of metabolites, but the presence of CGA isomers was not reported in either the plasma or the tissues [104,123,138,151].

FA glucuronide and FA sulfate were the metabolites found for FA, while CA, FA and *m-*COA were detected in the plasma for RA, as well as their glucuronidation, sulfation and methylation products [158,159,161,170]. Most of the FA was detected in its sulfated forms, which is also described for the human intestinal and liver S9 homogenates and Caco-2 cells, while FA glucuronide was the metabolite found in the highest concentration in HepG2 cells [101,109,121,122,123,127,158]. For RA, the ratio between the AUC of the glucuronidated/free RA forms was 3.2, indicating that more than 75% of RA in the bloodstream was metabolized, while its hydrolysis occurred to a lesser extent [159,161,162]. Indeed, the experiments in the Caco-2 cells, HepG2 cells and liver microsome showed the glucuronidation and hydrolysis of RA, but the concentration of metabolized RA was considerably lower than the free RA [122,124,161].

The metabolites found in the in vivo evaluations are qualitatively supported by the in vitro, ex vivo and in situ assays. The lack of one or other metabolite can be seen when comparing the studies, which can probably be related to the fact that full identification of the HCA metabolites was not the main objective of the studies.

#### 4.4.4. Elimination

Drug elimination is divided into two main processes: biotransformation and excretion. Biotransformation is the process in which the drug is converted to a metabolite, while excretion is the removal of the drug in the intact state or free form. The pharmacokinetic parameters that describe drug elimination are clearance, t_1/2_ and t_1/2β_ [165]. Based on these parameters, it is possible to affirm that the HCAs have a short elimination when orally administered (Table 2), which is consistent with their elevated distribution into elimination organs [152,158,163].

The rate of urinary excretion for free CA, *p*-COA and FA was higher in the first 6 h of urine collection, reaching 10.2%, 17.4% and 3%, respectively, after 48 h. CGA in its free form had a negligible urinary excretion of 0.04% in the same period [171]. On the other hand, RA was excreted more at the later collection points (8–18 h), showing a total rate of 0.44% [159]. Studies carried out for a reduced time (8–24 h) exhibited rate values even higher than the 48 h study for CA (9.9%), CGA (0.07–0.499%), *p*-COA (24%) and FA (5.4%) [136,150,151,163,172].

The renal clearance of CA (0.0157 L/h kg) and CGA (0.147–0.292 L/h kg) demonstrated that CA is eliminated basically by biotransformation, while the hepatic extraction ratio would be responsible for 23.1–28.2% of CGA elimination [146,150]. The concentration of HCA metabolites found in the urine was higher than their unchanged forms. The CA metabolites were structurally similar to those identified in the plasma [163]. For *p*-COA, its conjugated forms were detected without distinguishing types, while the RA metabolites recovered in urine were CA, CA glucuronide and sulfate, *m-*COA, *m-*COA glucuronide and sulfate, RA glucuronide and sulfate, methyl-RA, methyl-RA glucuronide and sulfate, FA, and FA glucuronide and sulfate [159,171]. 

Interestingly, even though the plasma concentration of free CGA was greater than its metabolites and it had low hepatic excretion, the ratio between the metabolites and the free form of CGA was 121, implying that CGA underwent renal metabolism. In addition, extra CGA metabolites were reported in the urine than in the plasma, such as free isoferulic acid, *m*- and *p*-COA, and 3-HPPA [68,150,151,171]. For FA, the urinary excretion of FA conjugates achieved a rate of 70.2%, of which FA glucuronide represented 5.1% [171,172]. 

The total urinary excretion, the sum of free and metabolized forms relative to the dose was higher for FA (73.2%) than CA (61.6%), *p*-COA (54.1%), RA (5.47%) and CGA (4.9%), demonstrating that FA was the most absorbed HCA, which is supported by the in vitro and ex vivo methods [159,171]. Nonetheless, its extensive biotransformation, illustrated by in situ and in vivo studies, decreases the bioavailable fraction, which is reflected in the low free concentration in plasma and urine. Taking only the free form of HCAs into account, *p-*COA exhibited the highest concentration in urine [68,171]. 

#### 4.4.5. Absolute Oral Bioavailability

The absolute oral bioavailability data demonstrated that *p*-COA was the most bioavailable HCA, followed by CA, RA and CGA (Table 2), in accordance with the rate of urinary excretion [171]. No data were found in the literature for FA; however, based on the results of the in situ and in vivo experiments, it possibly presents an absolute oral bioavailability intermediate to CA and RA. The in vivo data for RA were close to that described in the in vitro studies (Figure 3) [160]. However, the in vivo loss of RA after liver biotransformation was extremely high compared to the in vitro approach [122,161].

A decline in absolute oral bioavailability with the elevation of the dose was related to RA (Table 2). Analysis of the AUCs per dose revealed a lack of proportionality, indicating that RA has a non-linear pharmacokinetic feature [160]. The existence of dose proportionality was assumed for all doses tested for CGA, although a considerable reduction in renal clearance occurred with the increase in dose [150].

Some considerations are necessary with respect to the approach selected to analyze the pharmacokinetic profiles. Fourteen studies used a non-compartmental approach [75,105,147,148,149,150,151,152,154,157,158,159,160,161], while three reported a bicompartmental behavior [141,146,156]. The adoption of different analysis approaches produces misleading pharmacokinetic data, promoting the heterogeneity of results in the literature, which could hamper their use for clinical purposes [165]. Overall, the in vivo studies verified that the HCAs are quickly absorbed, and widely distributed, with physicochemical properties directly affecting their distribution, undergoing massive metabolization, and presenting fast elimination and low oral bioavailability.

## 5. Strategies to Improve Oral Bioavailability

Although out of the scope of this review, it is worth mentioning that recent reviews have gathered the technological strategies employed to improve the bioavailability of RA and FA, which are the most studied HCAs in this field. However, only a few studies have addressed their oral administration and evaluated the performance of the strategy in vivo [173,174,175]. 

Zhang et al. evaluated the effect of a nanostructured lipid carrier and solid lipid nanoparticles on the oral bioavailability of FA against its aqueous form, demonstrating that both formulations were able to improve the C_max_ and AUC of HCA. The in vivo performance of the nanostructured lipid carrier gave C_max_ and AUC parameters that were 1.32-fold and 1.38-fold better than solid lipid nanoparticles, a fact that was associated with the different release behavior presented for both formulations [157].

The incorporation of FA in a self-microemulsifying drug delivery system increased the AUC 1.73-fold when compared to its administration in an aqueous form. Additionally, it was observed that the emulsified FA had a higher body distribution, even in the brain [158]. 

The formulation of RA in a phospholipid complex and phospholipid complex oil solution promoted an increase of 47% and 225% in the C_max_ and 120% and 291% in the AUC, respectively, when compared with an aqueous RA solution [105]. Once again, the differences between the formulations were ascribed to the release, since the lower release of RA in the phospholipid complex oil solution decreased its pre-systemic metabolism and improved the systemic fraction.

Yang et al. demonstrated that the co-administration of RA with piperine, a glucuronidation inhibitor, increased the relative bioavailability of RA 1.24-, 1.32-, 2.02- and 2.26-fold in the presence of inhibitor at concentrations of 20, 40, 60 and 80 mg/kg, compared to the administration of RA [161].

With the exception of the data by Yang et al., [161] all technological strategies that have been explored to improve the oral bioavailability of FA and RA are lipid-based delivery systems. In general, these lipid-based systems promoted a higher bioavailability of HCAs by enhancing their stability in the gastrointestinal tract by modifying the release and improving the lipid solubility, facilitating their transport through the gastrointestinal mucosa and, to some extent, promoting lymphatic absorption [105,157,158].

Despite the available in vivo studies focusing only on one type of system, most of the technological approaches described in the reviews could be easily applied to oral administration, such as cyclodextrin complexes, solid dispersions, and polymeric nano and microparticles [173,174,175], demonstrating an unexplored field of research. 

## 6. Conclusions

CA and FA are the HCAs with the most data in the literature describing factors and processes related to oral bioavailability by different approaches, while only a few results can be found for the *m-* and *o-*COA isomers. The majority of investigations in recent pharmacokinetic studies have involved CGA, *p*-COA and RA.

The data described in this review allow us to affirm that water solubility is apparently not a limiting step for the oral absorption of HCAs, but more studies are needed to better understand the effect of this factor on their oral bioavailability. On the other hand, a substantial loss of HCA content was observed in the gastrointestinal environment, which decreases the fraction available for absorption.

In vitro absorption by passive and carrier-mediated transporters was inexpressive for most HCAs and was influenced by their physicochemical properties, highlighting that the permeation across gastrointestinal membranes is another issue for their absorption. In addition to the absorption process, in vitro, in situ, ex vivo and in vivo methods showed the great impact of pre-systemic metabolism on the bioavailable fraction. The sum of all these factors is responsible for the clear low absolute oral bioavailability values found.

It is important to mention that the in vitro, ex vivo, in situ and in vivo methods, in general, showed a qualitative correlation in their results; however, some discrepancies were identified quantitatively. Additionally, each approach presents limitations and variables, requiring extreme care when interpreting the data.

Overall, the results described in this review highlight the main factors/obstacles that impact the oral administration of HCAs and can be a guide to the development of delivery systems to overcome the issues of HCAs and produce a more successful therapy. Some recent reviews have shown several technological approaches that could be applied to the oral delivery of HCAs, but lipid-based delivery systems and their in vivo oral administration were the main focus of the investigation, revealing new potential opportunities. 

## Figures and Tables

**Table 2:** Pharmacokinetic parameters of orally administered HCAs.

HCA	Animal Species	Dose (mg/kg)	C_max_ (mg/L)	T_max_ (h)	t_1/2_ (h)	MRT_0→∞_ (h)	AUC_0→∞_ (mg h/L)	Vd (L/kg)	Cl (L/h kg)	AUC_0→∞_/Dose	F (%)	References
CA	Sprague-Dawley rats	120.0	-	-	absorption: 0.08 α: 0.14 β: 3.14	-	278.3	0.518	0.115	2.32	26.54	[146]
CA	Wistar rats	18.0	2.023 ^a^ 0.409 ^b^	0.17 ^a^ 0.17 ^b^	0.58 ^a^ 0.57 ^b^	- -	1.755 ^a,c^ 0.329 ^b,c^	- -	- -	0.0975 ^a,c^ 0.0183 ^b,c^	- -	[147]
CA	Sprague-Dawley rats	20.0	7.871	0.33	1.25	1.72	14.03	-		0.702	3.4	[148]
CA	Sprague–Dawley rats	10.0	0.25	0.33	β: 2.13	2.96	0.355	2.41	3.35	0.0355	14.7	[141]
CGA	Wistar rats	50.0	0.55	0.48	1.7	-	1.61	97.5	39	0.003	-	[149]
CGA	Sprague–Dawley rats	1.0 2.0 4.0 8.0	0.00245 0.00912 0.019 0.021	0.25 0.5 1.5 0.75	- - - -	- - - -	0.0078 ^c^ 0.017 ^c^ 0.041 ^c^ 0.065 ^c^	- - - -	0.618–0.726	0.0078 0.0085 0.0103 0.0081	0.478 0.522 0.718 0.569	[150]
CGA	Wistar rats	240.0	1.855	0.5	-	-	-	-	-	-	-	[151]
CGA	Kunming mices	1200.0	82.6	0.17	-	-	51.388 ^c^	-	-	0.0428	-	[152]
*p-*COA	Sprague-Dawley rats	16.4	27.17 ^a^ 16.29 ^b^	0.17 ^a^ 0.17 ^b^	0.27 ^a^ -	- -	8.176 ^a,c^ -	- -	- -	0.499 ^a^ -	- -	[153]
*p-*COA	Wistar rats	2.35	3.15	0.17	1.28	-	2.32	-	-	0.987	51.8	[75]
*p*-COA	Wistar rats	7.38	4.29	0.18	0.77	-	2.502	-	-	0.339	-	[154]
*p*-COA	Human	258 *	0.02195	0.5	0.9	-	0.2082	-	-	0.00008	-	[155]
FA	Sprague-Dawley rats	10.0	8.175	0.03	absorption: 0.07 α: 0.16 β: 1.77	-	2.962	-	-	0.296	-	[156]
FA	Sprague-Dawley rats	80.0	9.98	1.33	2.14	3.33	34.75	-	-	0.434	-	[157]
FA	Wistar rats	40.0	73.2	0.58	1.39	1.40 ^d^	82.86 ^c^	1.22 × 10^6^	2.5 × 10^5^	2.072	-	[158]
RA	Sprague-Dawley rats	50.0	1.667	0.5	-	-	-	-	-	-	-	[159]
RA	Wistar rats	36.0	0.489 ^a^ 0.166 ^b^	0.17 ^a^ 0.08 ^b^	0.95 ^a^ 1.1 ^b^	- -	0.3624 ^a,c^ 0.0996 ^b,c^	- -	- -	0.01 ^a,c^ 0.003 ^a,c^	- -	[147]
RA	Sprague-Dawley rats	50.0	0.327	0.33	6.77	-	1.395	-	-	0.028	-	[105]
RA	Sprague-Dawley rats	12.5 25.0 50.0	0.215 0.362 0.791	0.14 0.18 0.31	5.54 5.24 4.92	7.32 7.25 6.11	0.867 1.310 1.867	111.83 141.81 197.65	15 19.2 27.6	0.069 0.053 0.037	1.69 1.28 0.91	[160]
RA	Sprague-Dawley rats	50.0	0.416	0.15	5.02	-	1.246	-	-	0.025	-	[161]
RA	Sprague-Dawley rats	50.0	1.088	0.19	1.34	1.57	0.972	-	-	0.019	4.13	[162]

C_max_: maximum concentration. T_max_: maximum time. t_1/2_: half-life time. MRT: mean residence time. AUC: area under the curve. Vd: volume of distribution. Cl: clearance. F: absolute bioavailability. ^a^ Sample collected from portal vein. ^b^ Sample collected from abdominal artery. ^c^ AUC_0→t_ (mg h/L). ^d^ MRT_0→t_ (h). * Only mg.
